# Effect of Mineral Fillers on the Mechanical Properties of Commercially Available Biodegradable Polymers

**DOI:** 10.3390/polym13030394

**Published:** 2021-01-27

**Authors:** Wouter Post, Lambertus J. Kuijpers, Martin Zijlstra, Maarten van der Zee, Karin Molenveld

**Affiliations:** Wageningen Food and Biobased Research, Bornse Weilanden 9, 6708 WG Wageningen, The Netherlands; bert.kuijpers@wur.nl (L.J.K.); martin.zijlstra@wur.nl (M.Z.); maarten.vanderzee@wur.nl (M.v.d.Z.); karin.molenveld@wur.nl (K.M.)

**Keywords:** biodegradable polymer, soil-biodegradable, tensile properties, impact resistance, particulate polymer composites

## Abstract

In the successful transition towards a circular materials economy, the implementation of biobased and biodegradable plastics is a major prerequisite. To prevent the accumulation of plastic material in the open environment, plastic products should be both recyclable and biodegradable. Research and development actions in the past few decades have led to the commercial availability of a number of polymers that fulfil both end-of-life routes. However, these biobased and biodegradable polymers typically have mechanical properties that are not on par with the non-biodegradable plastic products they intend to replace. This can be improved using particulate mineral fillers such as talc, calcium carbonate, kaolin, and mica. This study shows that composites thereof with polybutylene succinate (PBS), polyhydroxybutyrate-hexanoate (PHBH), polybutylene succinate adipate (PBSA), and polybutylene adipate terephthalate (PBAT) as matrix polymers result in plastic materials with mechanical properties ranging from tough elastic towards strong and rigid. It is demonstrated that the balance between the Young’s modulus and the impact resistance for this set of polymer composites is subtle, but a select number of investigated compositions yield a combination of industrially relevant mechanical characteristics. Finally, it is shown that the inclusion of mineral fillers into biodegradable polymers does not negate the microbial disintegration of these polymers, although the nature of the filler does affect the biodegradation rate of the matrix polymer.

## 1. Introduction

The development of plastic materials in the 20th century has led to a multitude of plastic products that combine lightweight, high performing mechanical properties and outstanding barrier properties with the ability to be molded in virtually any shape imaginable. As a result, plastic products have become an indispensable part of human life as they are found in applications such as food packaging, clothing, home appliances, and toys. The downside of this innovation is that society now faces a huge challenge to properly dispose and recycle these plastic products and prevent the accumulation of plastics in the natural environment. Even though the need for a functional recycling infrastructure is clear, which is reflected in the development of new and improved waste collection, sorting, and recycling (e.g., pyrolysis) technologies, there is still an unacceptable large fraction of plastic waste that ends up in the natural environment [1]. As a result of their chemical composition, the majority of plastic waste originating from day-to-day products (mainly polyethylene (PE), polypropylene (PP), and polyethylene terephthalate (PET)) are not broken down by the microbes present in the respective environments. Therefore, it would be highly advantageous if plastic products that have a high risk of ending up in nature will be, aside from being recyclable, biodegradable in both soil and marine environments. In order to fulfil this demand, many academic initiatives on the development of biodegradable polymers have taken place in the past few decades. As some of these initiatives have proven to be very fruitful, there are a number of biodegradable polymers, such as poly (lactic acid) (PLA), polybutylene succinate (PBS), polybutylene succinate adipate (PBSA), polyhydroxy alkanoates (PHAs), and polybutylene adipate terephthalate (PBAT), that are currently being produced at a (semi-)industrial scale [2,3]. It has to be noted that biodegradability is considered to be a system property, resulting from the interaction between the material properties of the plastic and the biotic and abiotic conditions of the environment in which it biodegrades. Therefore the environment in which biodegradation occurs always needs to be taken into account [4]. As an example, PLA has a high biodegradation rate at temperatures well above room-temperature and is therefore very suitable for industrial compositing processes. However, even though its biodegradation rate in soil and aquatic environments is higher than that of PE, PP, and PET, it can still persist in these environments and thereby accumulate for multiple decades if it ends up there [5]. Other biodegradable polymers such as PBS and PBSA show biodegradation rates in soil environments that are several orders of magnitude higher than that of PE-, PP-, and PET-based plastics, although they are typically not certified as such since the timeframe does not always match the certification standards [6]. In this respect, PHA and PBAT polymers are amongst the few polymers that are (certified) biodegradable in all environments including marine conditions and therefore, show much potential for use in high littering risk applications [7,8]. The ability to process these biodegradable polymers in complex shapes required for products such as bags, containers, and toys is most often comparable to that of non-biodegradable plastics. However, a clear disadvantage is that the number of biodegradable polymers that possess both the stiffness and impact resistance that are required for these applications is limited [7,9]. This is one of the reasons that these biodegradable polymers are often not seen as acceptable alternatives for conventional non-biodegradable plastics. In addition, it has to be noted that currently, the high production costs prevent industry acceptance of these polymers. An often employed approach to obtain relevant polymer properties is by blending various biodegradable polymers via polymer extrusion. For example, many investigations were performed on PBS/PLA blended materials with the aim of obtaining plastic formulations with mechanical properties in the range of polypropylene (PP) [10,11,12]. Although this strategy allows for the optimization of mechanical properties, the biodegradable character of these immiscible blends will still be governed by the polymer with the lowest intrinsic biodegradation rate [13]. Another feasible approach to improve the mechanical properties is by distributing inorganic mineral fillers into the polymer matrix phase, thereby creating particulate polymer composites [14,15,16]. The addition of reinforcing filler material is a well-established strategy in both academia and industry. A disadvantage of this approach is the effect that the addition of mineral fillers has on the recyclability of these polymers, although this highly depends on the recycling infrastructure in which these materials end up. The effect of mineral filler reinforcement on the biodegradation end-of-life route is less detrimental as the release of these fillers upon biodegradation does not affect the overall concentrations that are typically already present in this environment [17]. A final advantage of the inclusion of particulate fillers in biodegradable polymers is that in general, the overall cost price of these materials is substantially lowered compared to unfilled polymers. Although the main academic focus of biodegradable polymer reinforcement in recent years has been on the use of organic natural fillers [18,19,20], the use of inorganic mineral fillers such as talc [21,22], calcium carbonate [23,24], kaolin [25,26], and mica [27,28] and their impact on the biodegradability of these polymers [29,30,31] has been investigated as well. As the properties of particulate polymer composites can highly fluctuate strongly, due to variations in additional polymer additives, processing techniques, and the applied test methods, it is generally complex to compare individually reported results [32,33,34]. Therefore, this work is a comparative study on the effect of various particulate fillers (talc, calcium carbonate, kaolinite, mica) on the most important mechanical properties of a selected number of industrially available biodegradable polymers (PBS, PBSA, PHBH, and PBAT) while keeping all other factors constant. Furthermore, an investigation is performed on the effect of particle addition on the biodegradability of these polymers. The resulting overview gives a clear overview of the possibilities to tailor the mechanical properties of biodegradable polymers towards specific plastic applications using particulate fillers.

## 2. Experimental

### 2.1. Materials

Polybutylene succinate (PBS) BioPBS FZ91PB and polybutylene succinate adipate (PBSA) BioPBS FD92PB were obtained from MCPP, Dusseldorf, Germany. Polybutylene adipate terephthalate (PBAT) Technipol 061/E was obtained from Sipol, Mortara, Italy. Poly(3-hydroxybutyrate-co-3-hydroxyhexanoate) (PHBH) Aonilex X331N was obtained from Kaneka, Westerlo-Oevel, Belgium. Talc Luzenac A10HC powder (d50% = 2.1 μm) and Mica 200k powder (d50% = 60 μm) were obtained from Imerys, Toulouse, France. Calcium carbonated chalk VS10 powder (d50% = 1.8 μm) was obtained from Omya, Oftringen, Switzerland. Kaolin Burgess Iceberg powder (d50% = 1.4 μm) was obtained from Burgess Pigment, Sanderville, GA, US.

### 2.2. Compound Preparation

All polymers were dried at 83 °C for at least 8 h in a Gerco two-chamber dry-air desiccant dryer (TTM 2/100 ES, Gerco Kunstofftechnik GmbH, Warendorf, Germany) to achieve moisture contents below 250 ppm prior to further processing. Extrusion was performed with a Berstorff ZE25x40D twin screw co-rotating extruder (KraussMaffei Berstorf GmBH, Hannover, Germany) at maximum temperatures of 180, 180, 160, and 180 °C for PBS, PBSA, PBAT, and PHBH, respectively. Injection molding was performed with a Sumitomo Demag IntElect 2 (Sumitomo Demag Plastics Machinery GmbH, Schwaig, Germany) at maximum temperatures of 170, 170, 160, and 180 °C for PBS, PBSA, PBAT, and PHBH, respectively. Tensile bars (75 mm × 5 mm × 2 mm) were produced conforming to ISO527-2 and impact bars (80 mm × 10 mm × 4 mm) were produced according to ISO 294. Sheet extrusion was performed with a Dr. Collin single screw extruder (COLLIN Lab & Pilot Solutions GmbH, Maitenbeth, Germany) with a sheet die at maximum temperatures of 180, 180, 135, and 175 °C for PBS, PBSA, PBAT, and PHBH, respectively.

### 2.3. Mechanical Characterization

Mechanical properties of the polymer compounds were evaluated using a Zwick Z010 tester with Multisens extenso meters (ZwickRoell, Venlo, The Netherlands), which was built according to ISO 527. Tests were performed following standards ISO 527-2 (for injection molded samples) and ISO 527-3 (for sheets and films). Measurements were performed in 5-fold. The test speed for determination of the Young’s modulus was 1 and 10 mm/min for determination of the strength at break and strain at break. Charpy impact resistance of the polymer compounds was evaluated using a Ceast 9050 tester (Instron, Boechout, Belgium) equipped with a Charpy 4J hammer which was built according to ISO 179 and conforming to the method based on the standards ISO 179-1eU (notched).

### 2.4. Physical Characterization

GPC measurements to determine molar mass were performed using a Viscotech VE 2001 GPC max provided with a TDA305 Triple Detector Array (RALLS + LALLS, RI Detector and Viscometer). Columns used were a PSS PFG analytical linear M and guard column, molecular range ~250–2.5∙10e6 D (PMMA in HFIP). The selected solvent was hexafluoroisopropanol (HFIP) with 0.02 M potassium trifluoroacetate (KTFA). All measurements were performed in duplicate. The Melt Flow Index (MFI) was determined using a Zwick MFlow tester according to ISO 1133 and conforming to the methods described in ASTM D1238 and ISO 1133.

### 2.5. Biodegradation Assessment

Assessment of the biodegradation in soil was based on the method described in ASTM G160-12(2019) [35].

Dumbbell-shaped samples were cut from sheets and were exposed to soil under laboratory-controlled conditions. The soil, a sandy topsoil obtained from organic experimental and training Farm “Droevendaal” (Part of Wageningen University and Research, NL), was inoculated with 2% (based on dry weight) fresh, mature compost from an industrial composting facility treating the organic fraction of municipal solid waste. Containers (40 cm × 16 cm × 18 cm) with buried samples were incubated at constant temperature (25 ± 2 °C) and relative humidity (90 ± 5%). Moisture content of the soil was maintained constant at 80% of the water holding capacity by spraying demineralized water to correct for any evaporation during the incubation. Viability of the soil was regularly checked with an untreated cotton cloth reference, which should lose >50% of the initial tensile strength in 5 days of exposure to the soil. In addition, soil pH was monitored as well.

Plastic samples were recovered from the soil at regular intervals; for this purpose, 6 replicate samples were buried per datapoint. Recovered samples were carefully rinsed and cleaned to remove the soil and subsequently photographed. Prior to mechanical analyses, the samples were conditioned for 1 week at 50% RH and 23 °C. Control samples were recovered after of 1 day of exposure to exclude any influence of the burial and cleaning procedure.

## 3. Results and Discussion

### 3.1. The Effect of Various Fillers on the Mechanical Properties of PBS

**Figure 1:**
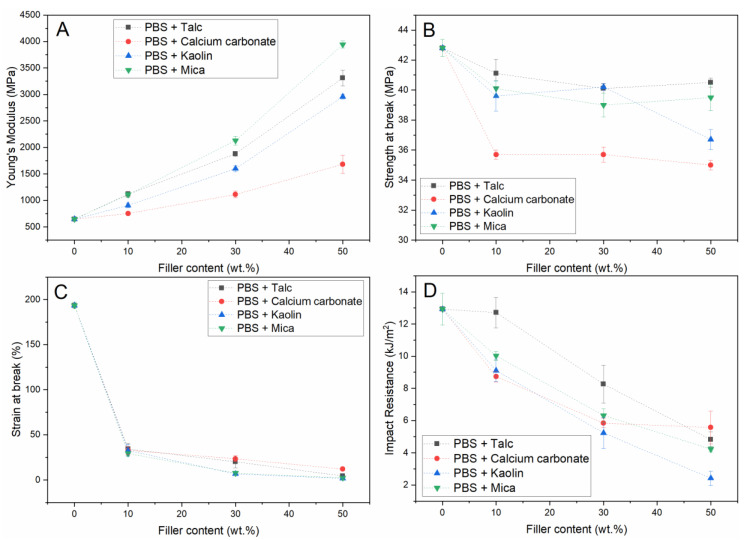
Mechanical properties (Young’s modulus (**A**), strength at break (**B**), strain at break (**C**), and notched impact resistance (**D**)) of 4 different fillers in PBS.

PBS was selected as one of the model matrices to compare the effect of different types of inorganic mineral fillers on the mechanical properties of biodegradable polymer composites. The non-reinforced polymer has properties in the same order of magnitude as PP, although the modulus is typically a factor 2–3 too low for adequate material substitution in current non-biodegradable applications. Furthermore, several applications benefit from an outstanding water vapor barrier capacity and optical properties, in which PP clearly outperforms PBS, but these properties are not included in this overview. The effect of talc, calcium carbonate, kaolin, and mica fillers on the selected mechanical properties (modulus, strength at break, strain at break, and notched impact resistance) of PBS is depicted in Figure 1. The fillers selected for this study all have an average particle size in the range of 2–20 μm. Aside from small variations in particle size, the main difference between the selected fillers lies in the particle shape. In this respect, calcium carbonate particles can be considered as near-spherical with an aspect ratio close to 1. Talc and kaolin are both plate-like particles with a substantially higher aspect ratio that can range up to 20 [36]. Finally, mica particles are also plate like particles that typically possess even higher aspect ratios that can go up to 100 [37].

Figure 1A shows that the modulus of the PBS gradually increases upon increasing filler content and that the addition of 10 wt.% mica and talc already yields a modulus that is two times higher than the unfilled matrix. Furthermore, the figure shows that upon increasing filler content, the highest moduli are obtained for mica-filled polymers, whereas the calcium carbonate composites yield lower values than all other tested compounds at equal filler content. This difference is mainly attributed to the difference in shape between these fillers as it is generally observed that a higher particle aspect ratio is accompanied with a stronger increase in Young’s modulus [38]. Figure 1B shows that the initial loading with filler material results in a moderate drop in tensile strength and that this effect is most profound for calcium carbonate-filled composites. However, the measured strength is unaffected by an additional increase in filler content which correlates with the initial drop in strain at break that is observed in Figure 1C. This figure shows that addition of any concentration of fillers is directly detrimental for the elasticity of these polymers as they act as stress concentration points, which is in correspondence with the effect generally observed in particulate-reinforced plastics. This fracture behavior also explains that a similar trend is observed for the impact resistance of notched test specimens that is shown in Figure 1D. Although the initial decline is not as steep as observed for the measured strain at break, it is shown that the particulate composites become substantially less tough with increasing filler content. It is noteworthy to address that talc outperforms the other minerals tested for low concentrations of filler and that an addition of up to 10 wt.% talc does not seem to negatively affect this property at all.

**Figure 2:**
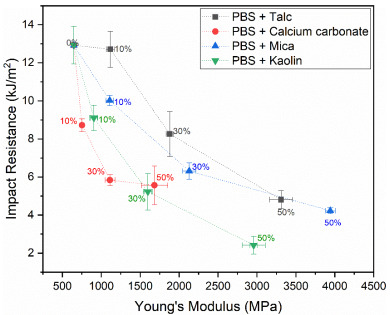
Relation between the Young’s modulus and notched impact resistance particulate PBS composites.

In polymer composite engineering (and material sciences in general), an increase in modulus is typically accompanied with a reduced impact resistance. In order to facilitate material selection, it is insightful to compare these properties to show the relation between these counteracting material properties comparable to the well-known Ashby diagrams [39]. Figure 2 shows this relation for the PBS composites developed in this study.

Based on the comparison in Figure 2, it becomes apparent that the talc–PBS composites outperform the other PBS materials studied in this work. When targeting the substitution of polypropylene (Young’s modulus of 1000–2000 MPa and impact resistance of 5–20 kJ/m^2^) in applications that mainly rely on mechanical performance, this figure indicates that PBS filled with 10–30 wt.% talc might be a suitable biodegradable alternative. Only when considering applications in which very high moduli are required, it might be favorable to select a different filler such as mica. At talc and mica filler concentrations around 50 wt.%, moduli and impact resistances in the range of PLA (3000–4000 MPa and 2–4 kJ/m^2^) are obtained. Based on this analysis, the resulting polymer composites could be considered as a PLA alternative that has a higher biodegradation rate in natural environments such as soil. However, this will depend on the targeted application as it is anticipated that complex processing operations such as thin-walled injection molding and sheet extrusion are severely complicated at these high filler ratios.

### 3.2. The Effect of Various Fillers on the Mechanical Properties of PHBH

**Figure 3:**
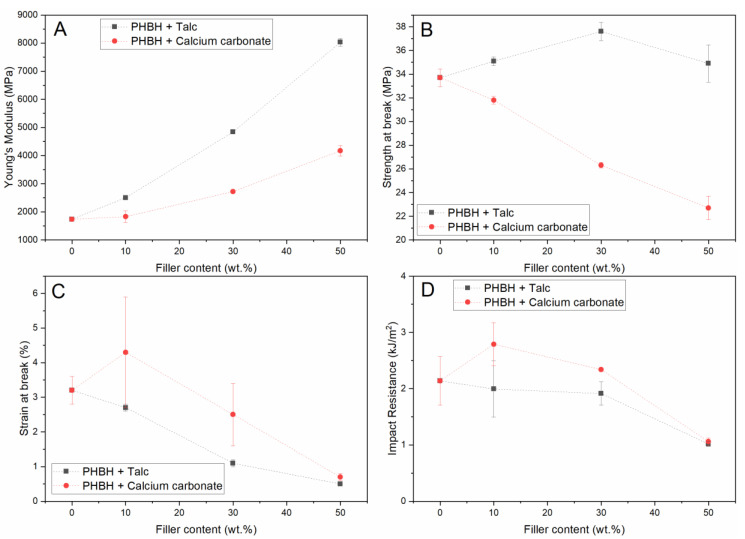
Mechanical properties (Young’s Modulus (**A**), strength at break (**B**), strain at break (**C**), and notched impact resistance (**D**)) of talc and calcium carbonate in PHBH.

An injection molding PHBH grade was selected as a second model polymer matrix due to the substantially different mechanical performance compared to PBS. In addition, the selected compound is certified marine biodegradable (OK biodegradable MARINE [40]). Figure 3 shows the mechanical performance of calcium carbonate and talc-filled composites for similar filler content as the PBS-based composites in Figure 1. As the results in Figure 2 show that talc and calcium carbonate PBS composites represent the upper and lower range of moduli and impact resistances, mica and kaolin were not included in the experiments with PHBH.

**Figure 4:**
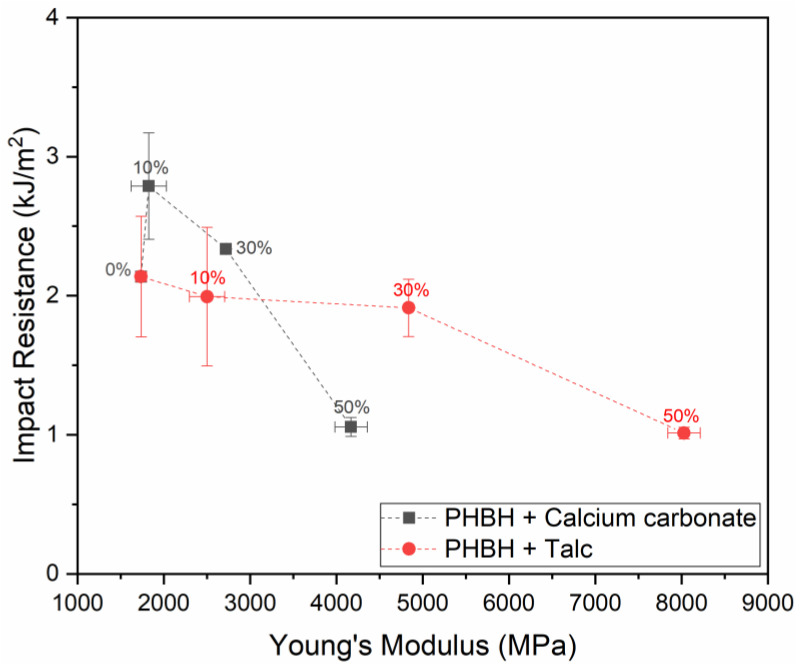
Relation between the Young’s modulus and notched impact resistance particulate PHBH composites.

Figure 3A shows that the modulus of PHBH is gradually increased upon the addition of both talc and calcium carbonate up to a modulus around 8000 MPa for a 50 wt.% talc-filled compound. These results are in accordance with the trends observed in Figure 1A. The effect of the selected fillers on the strength, depicted in Figure 3B, does show a different trend than observed for PBS in Figure 1B. Here, a moderate increase in strength is observed for talc-filled PHBH, while a reduction in strength was observed for PBS-based materials. At the same time, a clear reduction in strength is visible when the polymer is loaded with calcium carbonate which is in line with results obtained in PBS. Figure 3C shows that unfilled polymer is significantly less tough than PBS and non-biodegradable polymers such as PP and that the addition of high quantities of filler material promotes this behavior up to a level where the strain at break is lower than 1%. Figure 3D shows that an increased filler content has a less detrimental effect on the impact resistance than it has on the strain at break. A small increase in impact resistance can even be observed for the lower concentrations of calcium carbonate used in this study. This is also reflected in the graphical comparison between the notched impact resistance and Young’s modulus of the PHBH composites that are depicted in Figure 4.

Figure 4 shows clearly that the modulus of the PHBH compounds can be increased by the 30 wt.% addition of both talc and calcium carbonate without compromising the impact resistance. Furthermore, at this level of calcium carbonate loading, both the modulus and the impact resistance are increased. Nevertheless, these compounds remain very brittle compared to PBS, regardless of the selected filler content. This brittle character is one of the main challenges that has to be overcome before these materials can be seen as a truly feasible alternative for use in plastic products that are currently made from non-biodegradable plastics. On top of that, the narrow processing window of PHAs is considered an additional issue that complexes the industrial application of this class of materials in general. In this work, this is reflected by the difference in the values measured for the mass average molar mass and melt flow index (MFI) for both PBS and PHBH that are shown in Table 1. Here, it is shown that compounding of PHBH composites via extrusion yields a clear reduction in molecular weight and increase in MFI which both indicate that polymer degradation has taken place during the processing operations. The degradation during processing of PHBH and PHAs in general is typically attributed to the molecular chain scission that is reported to occur at temperatures over 160 °C [41]. Furthermore, the addition of high quantities of filler increases the overall shear stress during the extrusion process which can also contribute to the observed degradation in this work. The PBS composites, which were subjected to a comparable process, possess a somewhat higher mass average molar mass and a reduced melt flow index compared to the unfilled PBS, which is in line with the traditional behavior of particulate-filled plastics. The increase in mass average molar mass of the PBS composites is attributed to a secondary crosslinking reaction that is more commonly observed in this type of compounds.

### 3.3. The Effect of Talc on the Mechanical Properties of Various Biodegradable Polymers

**Figure 5:**
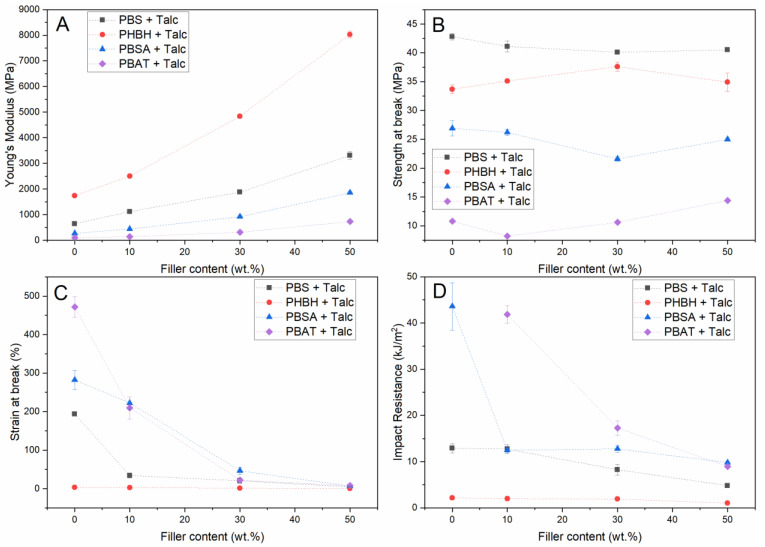
Mechanical properties (Young’s modulus (**A**), strength at break (**B**), strain at break (**C**), and notched impact resistance (**D**)) of talc-filled PBS, PHBH, PBSA, and PBAT.

Figure 1 and Figure 3 show that the impact of talc on the mechanical properties is more substantial than the other mineral fillers investigated in this work. Besides investigating the effect on PBS and PHBH matrices, the effect of talc on the mechanical properties of a broader selection of biodegradable polymers was studied. Figure 5 shows the effect of different talc concentrations on the mechanical properties of PBS, PHBH, PBSA, and PBAT.

**Figure 6:**
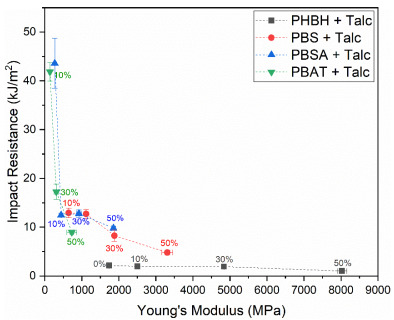
Relation between the Young’s modulus and notched impact resistance of talc-filled PBS, PBSA, PHBH, and PBAT composites.

As PBSA and PBAT are less rigid polymers than PHBH and PBS, they possess lower moduli and strength and a higher strain at break and impact resistance in their unfilled form. Figure 5A,B show that the effect of talc on the modulus and ultimate strength of PBSA and PBAT corresponds to the trends that were already observed for PBS and PHBH. Figure 5C shows that the strain at break evolution upon increased filler content for PBSA and PBAT also compares to the trends observed for PBS and PHBH, although the higher initial strain of the base polymers makes that the addition of 10 wt.% is not directly detrimental for this mechanical characteristic. The reported strain values (±200%) are well within the specifications that are considered acceptable for the majority of plastic applications. A further increase in talc content reduces the strain at break towards levels that are in the same order of magnitude as PBS composites with a similar talc loading. These composites could still be applicable for plastic products that do not require severe levels of strain in use (e.g., rigid food packaging, home appliances, and toys). Figure 5D shows that the talc-filled PBAT composites outperform all other compounds developed in this study when it comes to impact resistance. This is in accordance with the superior impact resistance of the unfilled polymer which is not included in Figure 5D as the applied testing method did not break the sample and therefore, no representative value could be registered. Therefore, the unfilled PBAT value is also not included in Figure 6, in which the Young’s moduli and impact resistance of all talc-filled composites are graphically compared. This figure clearly shows that the balance between these two mechanical characteristics is very subtle as a small increase in one can have a severe impact on the other. The figure also shows that when targeting PP-like mechanical properties (modulus of 1000–2000 MPa and impact resistance of 5–20 kJ/m^2^), several of the composites developed in this work could yield a biodegradable alternative.

### 3.4. The Effect of Talc and Calcium Carbonate on the Biodegradation of PHBH in Soil

**Figure 7:**
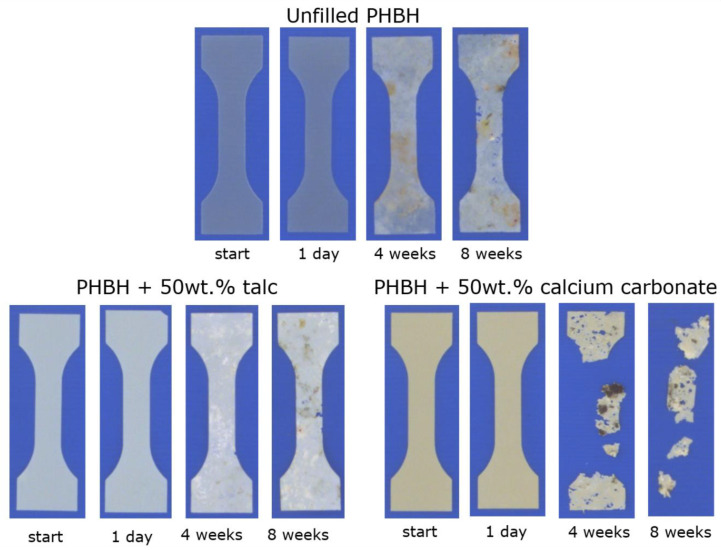
Visual evaluation of biodegradation in soil of unfilled (sheet thickness = 500 μm) and talc- and calcium carbonate-filled PHBH (sheet thickness = 200 μm).

The polymers investigated in this study all show biodegradation rates in soil that are substantially higher than those of conventional non-biodegradable polyolefins (e.g., PE/PP) and polyesters (e.g., PET). This material property gives these polymers an additional end-of-life scenario that can come into effect when products made from these materials intentionally or unintentionally end up in the natural environment. However, as it shown in this study that the use of mineral fillers can be used to direct their mechanical properties towards those of industrially applied plastics, it is crucial to identify whether these additives do not negate their unique biodegrading features. The full characterization of polymer composite biodegradability requires monitoring of the actual demineralization process into water and carbon dioxide, which is beyond the scope of this study. Nevertheless, a soil disintegration test was performed to identify the effect of both calcium carbonate and talc on the soil biodegradation of PHBH. The visual disintegration of three PHBH compounds upon soil exposure in a timeframe of 8 weeks is depicted in Figure 7. This figure shows that unfilled PHBH shows local patches of discoloration that are attributed to microbial activity within the first 4 weeks of the experiment, although no apparent loss of material is visible at this point of measurement. This discoloration is not simply attributed to the handling and soil staining of the test samples as the specimens that were exposed for only 1 day do not show this effect. Upon soil exposure of 8 weeks, these specimens start to show the first signs of visible disintegration, which is reflected by the presence of visible defects in the polymer sheet which are also attributed to microbial digestion.

**Figure 8:**
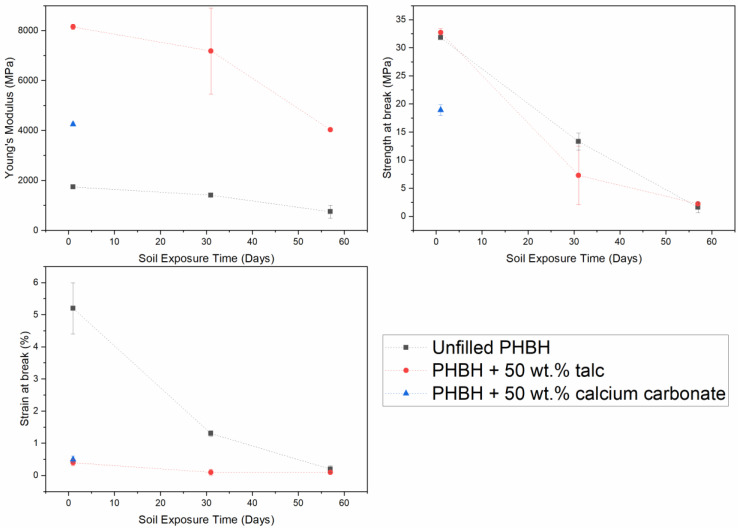
Effect of biodegradation in soil on the mechanical properties of calcium carbonate-/talc-filled PHBH.

Aside from the visual observations in Figure 7, the recovered test specimens were subjected to a tensile test in order to assess the decay of the modulus, strength at break, and strain at break over time. These results are shown in Figure 8. Here, it is shown that all mechanical properties of the unfilled PHBH specimens are gradually lowered as the soil exposure time is increased, which corresponds with the visual observations of Figure 7.

Figure 7 also shows that the disintegration of the talc-filled PHBH specimens follows a similar trend as was observed for the unfilled specimens with discoloration after 4 weeks and the manifestation of visible defects after 8 weeks. This visual disintegration is supported by the observed decay in mechanical properties that is shown in Figure 8. Based on this analysis, it could be argued that the addition of high quantities of talc does not impact the biodegradation rate of the polymer. However, the fact that the thickness of the unfilled specimens was 2.5 times higher due to different sheet processing parameters and the earlier addressed thermal degradation took place in the composite specimen means that this conclusion cannot be drawn based on these results. Nevertheless, based on these observations, it can be stated that talc–PHBH composites maintain their biodegradable character and thereby their unique end-of-life scenario compared to non-biodegradable plastics. The fact that during a final measurement point after 16 weeks, no substantial test specimen for any of the tested compounds could be recovered from the soil due to severe disintegration reinforces this statement. A similar conclusion can be drawn for the calcium carbonate–PHBH composites as the observed visual disintegration rates are significantly higher than those of their talc–PHBH counterparts. This rapid degradation also led to the fact that the test specimens could no longer be measured by means of tensile testing which is why Figure 8 only shows the properties of the calcium carbonate–PHBH composite after 1 day of soil exposure. In addition, the observed degradation rate is also higher than that of the unfilled PHBH. However, the difference in sample dimension and preparation means that the claim that the addition of calcium carbonate accelerates the biodegradation of PHBH is unfound. Still, the difference that is observed between the talc and calcium carbonate composites seems to indicate that the type of filler does affect the biodegradable characteristics of the overall composite. The fact that calcium carbonate–PHBH composites had a higher starting molecular weight than the talc–PHBH composites (Table 1) prior to soil exposure strengthens this observation. A potential explanation for this phenomenon could be that the alkaline nature of the calcium carbonate fillers prevents the acidification of the soil that is expected during the microbial depolymerization of PHBH polymers, but this has not been further quantified in this work.

## 4. Conclusions

This study gives a comprehensive overview of the effect of various particulate mineral fillers (talc, calcium carbonate, kaolin, and mica) within PBS, PHBH, PBSA, and PBAT polymers. Thereby, plastic materials with mechanical properties ranging from tough elastic towards strong and rigid are obtained. It is indicated that the balance between the Young’s modulus and the impact resistance for this set of polymer composites is subtle. Still, a select number of compounds yield a combination of industrially relevant mechanical characteristics. Finally, it is shown that the inclusion of mineral fillers into biodegradable polymers does not negate the microbial disintegration of these polymers and that the nature of the filler does affect the biodegradation rate of the matrix polymer.

## Figures and Tables

**Table 1:** Overview of mass average molar mass and melt flow index values of PBS and PHBH with 50 wt.% talc and calcium carbonate content.

Compound	Mass Average Molar Mass (kg/mol)	Melt Flow Index (g/10 min)
PBS	124	3.5
PBS + 50 wt.% talc	137	1.8
PBS + 50 wt.% calcium carbonate	172	0.5
PHBH	283	16.4
PHBH + 50 wt.% talc	140	79.4
PHBH + 50 wt.% calcium carbonate	183	74.6

## Data Availability

The data presented in this study are available on request from the corresponding author.

## References

[1] Van Velzen E.T., Jansen M., Brouwer M.T., Feil A., Molenveld K., Pretz T. Efficiency of recycling post-consumer plastic packages. Proceedings of the 32th International Conference of the Polymer Processing Society.

[2] Van den Oever M., Molenveld K. (2017). Replacing fossil based plastic performance products by bio-based plastic products—Technical feasibility. New Biotechnol..

[3] Iles A., Martin A.N. (2013). Expanding bioplastics production: Sustainable business innovation in the chemical industry. J. Clean. Prod..

[4] Van der Zee M. (2020). Methods for evaluating the biodegradability of environmentally degradable polymers. Handbook of Biodegradable Polymers.

[5] Lunt J. (1998). Large-scale production, properties and commercial applications of polylactic acid polymers. Polym. Degrad. Stab..

[6] Puchalski M., Szparaga G., Biela T., Gutowska A., Sztajnowski S., Krucińska I. (2018). Molecular and Supramolecular Changes in Polybutylene Succinate (PBS) and Polybutylene Succinate Adipate (PBSA) Copolymer during Degradation in Various Environmental Conditions. Polymers.

[7] Barron A., Sparks T.D. (2020). Commercial Marine-Degradable Polymers for Flexible Packaging. iScience.

[8] Dilkes-Hoffman L.S., Lant P.A., Laycock B., Pratt S. (2019). The rate of biodegradation of PHA bioplastics in the marine environment: A meta-study. Mar. Pollut. Bull..

[9] Siracusa V., Lotti N., Munari A., Rosa M.D. (2015). Poly(butylene succinate) and poly(butylene succinate-co-adipate) for food packaging applications: Gas barrier properties after stressed treatments. Polym. Degrad. Stab..

[10] Bhatia A., Gupta R.K., Bhattacharya S.N., Choi H.J. (2007). Compatibility of biodegradable poly (lactic acid) (PLA) and poly (butylene succinate) (PBS) blends for packaging application. Korea Aust. Rheol. J..

[11] Barletta M., Aversa C., Puopolo M., Donninelli A. (2019). Effect of micro-lamellar talc on dimensional accuracy and stability in injection molding of PLA/PBSA blends. Polym. Plast. Technol. Mater..

[12] Gigante V., Coltelli M.-B., Vannozzi A., Panariello L., Fusco A., Trombi L., Donnarumma G., Danti S., Lazzeri A. (2019). Flat die extruded biocompatible poly(lactic acid) (PLA)/poly(butylene succinate) (PBS) Based Films. Polymers.

[13] Yang S., Madbouly S.A., Schrader J.A., Srinivasan G., Grewell D., McCabe K.G., Kessler M.R., Graves W.R. (2015). Characterization and biodegradation behavior of bio-based poly(lactic acid) and soy protein blends for sustainable horticultural applications. Green Chem..

[14] Post W., Bose R.K., Garcia S.J., Van Der Zwaag S. (2016). Healing of Early Stage Fatigue Damage in Ionomer/Fe_3_O_4_ Nanoparticle Composites. Polymers.

[15] Zhong N., Post W. (2015). Self-repair of structural and functional composites with intrinsically self-healing polymer matrices: A review. Compos. Part A Appl. Sci. Manuf..

[16] Armentano I., Puglia D., Luzi F., Arciola C.R., Morena F., Martino S., Torre L. (2018). Nanocomposites Based on Biodegradable Polymers. Materials.

[17] Brebu M. (2020). Environmental Degradation of Plastic Composites with Natural Fillers—A Review. Polymers.

[18] Pivsa-Art S., Pivsa-Art W. (2021). Eco-friendly bamboo fiber-reinforced poly(butylene succinate) biocomposites. Polym. Compos..

[19] La Mantia F.P., Morreale M. (2011). Green composites: A brief review. Compos. Part A Appl. Sci. Manuf..

[20] Saba N., Tahir P.M., Jawaid M. (2014). A review on potentiality of nano filler/natural fiber filled polymer hybrid composites. Polymers.

[21] Raquez J.M., Nabar Y., Narayan R., Dubois P. (2008). Novel High-Performance Talc/Poly[(butylene adipate)-co-terephthalate] Hybrid Materials. Macromol. Mater. Eng..

[22] Sun B., Chuai C., Luo S., Guo Y., Han C. (2014). Effect of different amounts of modified talc on the mechanical, thermal, and crystallization properties of poly(butylene succinate). J. Polym. Eng..

[23] Chen R.-Y., Zou W., Zhang H.-C., Zhang G., Yang Z.-T., Jin G., Qu J.-P. (2015). Thermal behavior, dynamic mechanical properties and rheological properties of poly(butylene succinate) composites filled with nanometer calcium carbonate. Polym. Test..

[24] Nunes E.D.C., de Souza A.G., Rosa D.D.S. (2020). Use of a chain extender as a dispersing agent of the CaCO_3_ into PBAT matrix. J. Compos. Mater..

[25] Kwaśniewska A., Chocyk D., Gładyszewski G., Borc J., Świetlicki M., Gładyszewska B. (2020). The influence of kaolin clay on the mechanical properties and structure of thermoplastic starch films. Polymers.

[26] Gu L., Zhang S., Li H., Sun J., Tang W., Zhao L., Gu X. (2019). Preparation of Intumescent Flame Retardant Poly(butylene succinate) Using Urea Intercalated Kaolinite as Synergistic Agent. Fibers Polym..

[27] Sinha Ray S., Okamoto M. (2003). Polymer/layered silicate nanocomposites: A review from preparation to processing. Prog. Polym. Science.

[28] Zhang X., Lin G., Abou-Hussein R., Hassan M.K., Noda I., Mark J.E. (2007). Some novel layered-silicate nanocomposites based on a biodegradable hydroxybutyrate copolymer. Eur. Polym. J..

[29] Misra S.K., Valappil S.P., Roy A.I., Boccaccini A.R. (2006). Polyhydroxyalkanoate (PHA)/inorganic phase composites for tissue engineering applications. Biomacromolecules.

[30] Tolga S., Kabasci S., Duhme M. (2021). Progress of disintegration of polylactide (PLA)/poly(butylene succinate) (PBS) blends containing talc and chalk inorganic fillers under industrial composting conditions. Polymers.

[31] Qi R., Jones D.L., Liu Q., Liu Q., Li Z., Yan C. (2021). Field test on the biodegradation of poly(butylene adipate-co-terephthalate) based mulch films in soil. Polym. Test..

[32] Van den Oever M., Molenveld K. (2019). Creep deflection of Wood Polymer Composite profiles at demanding conditions. Case Stud. Constr. Mater..

[33] Ramos M., Dominici F., Luzi F., Jiménez A., Garrigós M.C., Torre L., Puglia D. (2020). Effect of almond shell waste on physicochemical properties of polyester-based biocomposites. Polymers.

[34] Mazur K., Jakubowska P., Romańska P., Kuciel S. (2020). Green high density polyethylene (HDPE) reinforced with basalt fiber and agricultural fillers for technical applications. Compos. Part B Eng..

[35] International A. (2019). ASTM G160—12(2019) Standard Practice for Evaluating Microbial Susceptibility of Nonmetallic Materials by Laboratory Soil Burial.

[36] Mallick P.K., Kelly A., Zweben C. (2000). 2.09—Particulate and Short Fiber Reinforced Polymer Composites, in Comprehensive Composite Materials.

[37] Kauffman S., Leidner J., Woodhams R., Xanthos M. (1974). The preparation and classification of high aspect ratio mica flakes for use in polymer reinforcement. Powder Technol..

[38] Leong Y.W., Abu Bakar M.B., Ishak Z.M., Ariffin A., Pukanszky B. (2004). Comparison of the mechanical properties and interfacial interactions between talc, kaolin, and calcium carbonate filled polypropylene composites. J. Appl. Polym. Sci..

[39] Ashby M. (2016). Materials Selection in Mechanical Design.

[40] Tüv Austria Belgium (2019). OK biodegradable MARINE. Bio Products—Degradation in Seawater.

[41] Xiang H., Wen X., Miu X., Li Y., Zhou Z., Zhu M. (2016). Thermal depolymerization mechanisms of poly(3-hydroxybutyrate-co-3-hydroxyvalerate). Prog. Nat. Sci. Mater. Int..

